# Bioenergetic Model of Retrotransposon Activity in Cancer Cells

**DOI:** 10.3390/life15091338

**Published:** 2025-08-23

**Authors:** Sergei Pavlov, Maria Duk, Vitaly V. Gursky, Maria Samsonova, Alexander Kanapin, Anastasia Samsonova

**Affiliations:** 1 Mathematical Biology and Bioinformatics Laboratory, Peter the Great Saint Petersburg Polytechnic University, Saint Petersburg 195251, Russia; sergpav92@mail.ru (S.P.); m.samsonova@spbstu.ru (M.S.); 2 Theoretical Department, Ioffe Institute, Saint Petersburg 194021, Russia; duk@mail.ioffe.ru; 3 Institute for Translational Biomedicine, St. Petersburg State University, Saint Petersburg 199034, Russia; a.kanapin@spbu.ru (A.K.); a.samsonova@spbu.ru (A.S.)

**Keywords:** retrotransposons, bioenergetic model, energy balance, cell death

## Abstract

Retrotransposons exhibit increased activity in cancer cells. One possible approach to anticancer therapy is to use this activity to influence the energy balance in cells. Abnormal distribution of retrotransposons in the genome requires additional energy consumption, which can lead to a significant decrease in the total amount of free ATP molecules in the cell. A decrease in ATP levels below a certain threshold can in turn trigger a cell death program. To investigate the possibility of such a scenario, we developed a mathematical model of the cellular energy balance that describes the dynamics of energy consumption by the main cellular processes, including costs of retrotransposon activity. The model considers changes in the concentrations of ATP, active retrotransposons (LINE-1 and SINE) in the human genome, as well as mRNAs and proteins that are expression products of retrotransposon and constitutive genes. We estimated the parameter values in the model based on literature data and numerical optimization. We found a single stable stationary solution, characterized by low retrotransposon activity, and used it as the reference steady state for further analysis. Parametric sensitivity analysis revealed the parameters whose changes had the greatest impact on cellular ATP levels. The LINE-1 deactivation rate constant and the maximum LINE-1 transcription rate were the most sensitive among the transposon-related parameters. Perturbation of these parameters led to a decrease in the number of free ATP to 30% of the reference value and below. Transcription of retrotransposons under perturbed parameters became comparable to the translation of constitutive genes in terms of energy costs. The presented results indicate that cancer cell death can be initiated by increasing the load on the energy balance due to the activation of transposons.

## 1. Introduction

Mobile genetic elements, or transposons, are DNA fragments that are capable of copying themselves to new positions [1]. They account for up to 45% of the human genome, but only a small fraction are currently capable of self-replication under normal conditions [2]. An elevated transposon replication activity may cause numerous negative cellular effects, including deleterious gene mutations, genomic instability, and oncogene activation, leading to the development and progression of human cancer [3]. Cells avoid these negative effects by activating transposon suppression mechanisms, including methylation and chromatin remodeling [4].

Retrotransposable elements (RTEs), or retrotransposons, are the only type of transposons that is currently active in the human genome. Their insertion rate is estimated to be between 1 in 20 and 1 in 900 births [5]. The most frequent RTEs include the long interspersed element (LINE-1, or L1) and short interspersed element (SINE) Alu. L1 is an autonomous RTE, which means that it codes for a protein (ORF2p) that forms part of the reverse transcriptase used to insert new copies of L1 into the genome. Nonautonomous Alu does not code for proteins but uses the transcriptase machinery produced by L1 for transposition. The competition for ORF2p between the two RTEs makes them evolve as a predator–prey dynamical system, in which Alu functions as a predator that restrains L1 activity and, thus, stabilizes L1 abundance in the genome [6].

RTEs demonstrate an increased activation in cancer cells, providing a significant oncogenic factor [7,8]. Unraveling the transposon regulation mechanisms in cancer cells is a promising direction in studies of human carcinogenesis [9]. Immunotherapy strategies for various pathologies use a mechanism associated with the interferon response to RTE activity in cells [10,11,12,13]. On the other hand, RTEs can also mediate cancer resistance and serve as tumor suppressors. The elevated RTE expression in premalignant cells activates cGAS-STING pathway to induce cell death [14]. L1 can be sensed as a DNA damaging event inducing apoptosis in cancer cells [15,16]. Therefore, controlling RTE activity in cancer cells to trigger cell death programs may be a promising approach to anticancer therapy.

We present a new approach to the RTE-mediated anticancer therapy based on the study of the changing energy balance in the cell experiencing an increased RTE expression. Cancer cells can demonstrate high plasticity of the energy balance, for example, by switching their metabolism to active glycolysis (the Warburg effect) [17,18,19]. This indicates the ability of cells to adapt to different levels of free energy, expressed, for example, in the amount of available ATP molecules. However, a moderate (about three-fold) decrease in this amount can initiate cell death programs [20,21,22]. Under such conditions, abnormally active transposons in cancer cells can actively compete with other cellular agents for available amounts of ATP and thereby shift the intracellular energy balance towards irreversible cell death. A high amount of active RTEs in the cell can be sustained or even initiated by either genetic editing or perturbing the rates of processes that promote new RTE insertions. The latter include silencing or RTE transcription and translation.

In this study, we investigated the theoretical feasibility of the described RTE-mediated energy depletion scenario. We developed a kinetic model of the cellular energy balance and explored possible stationary and dynamical regimes in this model. The model accounted for the main energy-consuming cellular processes, including those associated with the L1 and Alu activity. The model was parameterized using literature data on relevant parameter values for human cells, with an additional optimization for less well-known parameters. We analyzed the model by focusing on solutions with sufficiently low amounts of cellular ATP resulted from the increased RTE activity, considering such solutions as a theoretical ‘proof of concept’ for the idea of using controlled RTE activity to trigger cancer cell death programs.

## 2. Materials and Methods

**Figure 1:**
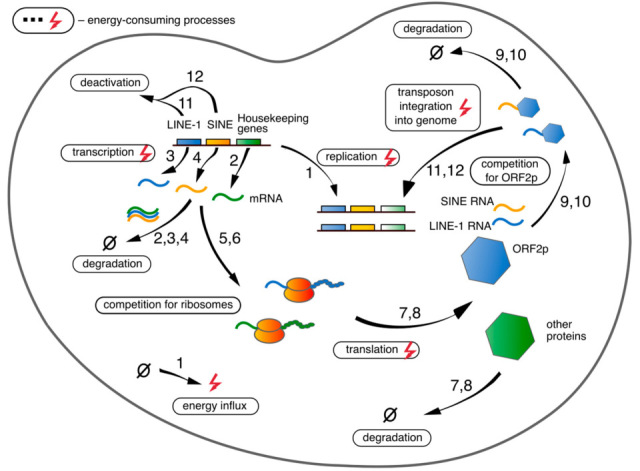
Cellular processes considered in the model. The numbers next to each arrow indicate the numbers of equations in model (1)–(12) which involve the corresponding processes.

We developed a model of the energy balance in a eukaryotic cell that incorporated the main cellular processes participating in energy consumption (Figure 1). Our focus was on investigating possibilities for RTEs to shift the balance, so the model also comprised processes associated with the RTE lifecycle. The energy available in the cell is represented in the model via the number of free ATP molecules (*a*), whose dynamics result from the balance between the constant influx and the consumption by cellular processes. The energy is spent on the transcription and translation of RTEs and other genes, integration of new RTE copies into the genome, and DNA replication. Table 1 presents all dynamical variables considered in the model.

In what follows, we describe the model equations (Section 2.1), all reaction rates underlying the model equations (Section 2.2), parameter values (Section 2.3), and details on numerical simulations (Section 2.4).

### 2.1. Model Equations

The bioenergetic model consists of the following ordinary differential equations representing the concentration dynamics of free cellular ATP, LINE-1 and SINE RTEs, mRNAs and proteins of RTEs and housekeeping genes, and various intermediate complexes:

(1) $$\frac{da}{dt}=A_{0}-\lambda_{a}a-\;v_{repl}\left(a,bL,O1,bS\right)-\;N_{nt}\left(N_{Q}\omega_{q}\left(a\right)+N_{L}\omega_{L}\left(L,a\right)+N_{S}\omega_{S}\left(S,a\right)\right)-\;N_{aa}(N_{q}v_{q}\left(cq,a\right)+\;\frac{N_{L}}{3}v_{L}\left(cL,a\right))-\;N_{nt}\left(N_{L}v_{\mathrm{int}L}\left(bL,O1,a\right)+N_{S}v_{\mathrm{int}S}\left(bS,a\right)\right)$$

(2) $$\frac{dmq}{dt}=\omega_{q}\left(a\right)-k_{bq}f_{rib}(cq,cL)mq+k_{uq}cq+v_{q}\left(cq,a\right)-d_{mq}mq$$

(3) $$\frac{dmL}{dt}=\omega_{L}\left(L,a\right)-k_{bL}f_{rib}\left(cq,cL\right)mL+k_{uL}cL+k_{\mathrm{sub}S}mS\;bL-k_{\mathrm{sub}L}mL\;bS-d_{mL}mL$$

(4) $$\frac{dmS}{dt}=\omega_{S}\left(S,a\right)-k_{\mathrm{sub}S}mS\;bL+k_{\mathrm{sub}L}mL\;bS-d_{mS}mS$$

(5) $$\frac{dcq}{dt}=k_{bq}f_{rib}\left(cq,cL\right)mq-k_{uq}cq-v_{q}\left(cq,a\right)-d_{cq}cq$$

(6) $$\frac{dcL}{dt}=k_{bL}f_{rib}\left(cq,cL\right)mL-k_{uL}cL-v_{L}\left(cL,a\right)-d_{cL}cL$$

(7) $$\frac{dq}{dt}=v_{q}\left(cq,a\right)-d_{q}q$$

(8) $$\frac{dO1}{dt}=v_{L}\left(cL,a\right)-v_{\mathrm{int}L}\left(bL,O1,a\right)-d_{O1}O1$$

(9) $$\frac{dbL}{dt}=v_{L}\left(cL,a\right)-v_{\mathrm{int}L}\left(bL,O1,a\right)-k_{\mathrm{sub}S}mS\;bL+k_{\mathrm{sub}L}mL\;bS-d_{bL}bL$$

(10) $$\frac{dbS}{dt}=k_{\mathrm{sub}S}mS\;bL-k_{\mathrm{sub}L}mL\;bS-v_{\mathrm{int}S}\left(bS,a\right)-d_{bS}bS$$

(11) $$\frac{dL}{dt}=v_{\mathrm{int}L}\left(bL,O1,a\right)-\lambda_{L}L$$

(12) $$\frac{dS}{dt}=v_{\mathrm{int}S}\left(bS,a\right)-\lambda_{S}S$$

Equation (1) describes the concentration dynamics of cellular ATP (*a*), which is supplied within the cell with a constant rate *A*_0_. All the other terms in the equation represent energy consumption. The second term describes ATP degradation with a rate constant *λ_a_*. The next term describes the DNA replication rate $v_{repl}$. The next three brackets describe the rates of the following energy-consuming processes: transcription of housekeeping genes ($\omega_{q}$) and transposons ($\omega_{L}$ and $\omega_{S}$ for L1 and Alu, respectively), translation of housekeeping genes’ transcripts ($v_{q}$) and L1′s RNAs ($v_{L}$), and integration of new transposon copies into the genome ($v_{\mathrm{int}L}$ and $v_{\mathrm{int}S}$). All reaction diagrams and reaction rates in the model are defined in Section 2.2. The factors at the rates in these brackets account for various numbers of reaction-specific molecules involved in the ATP consumption, as described in Table 2. In the model, we consider housekeeping genes as representatives of the transcribing part of DNA that participates in competition with RTEs for cellular ATP via its expression products.

Equation (2) describes the concentration dynamics of mRNAs coded by housekeeping genes (*mq*). The right-hand side of the equation contains the rates of the following processes (from left to right): transcription, mRNA-ribosome complex formation, mRNA unbinding from the mRNA-ribosome complexes, mRNA release at the finish of translation, and degradation. mRNAs bind to free ribosomes *r* forming complexes *cq*. The rate of this reaction follows the mass action kinetics with rate constant *k_bq_*. The concentration of free ribosomes reads as *r* = f_rib_(*cq*, *cL*) = *r*_tot_ − *cq* − *cL*, where *r*_tot_ is the total concentration of ribosomes (Table 2), and *cL* is the concentration of complexes consisting of ribosomes and mRNAs coded by L1. The unbinding of mRNAs from ribosomes also follows the mass action kinetics with rate constant *k_uq_*. Translation produces proteins *q* and releases mRNAs from the mRNA-ribosome complex, proceeding with rate *v_q_*. The last term in the equation describes degradation of housekeeping gene mRNAs with rate constant *d_mq_*.

Equations (3) and (4) describe the concentration dynamics of mRNAs coded by L1 (*mL*) and Alu (*mS*), respectively. The RTEs transcribe with rates *ω_L_*(*L*, *a*) and *ω_S_*(*S*, *a*), which depend on the number of active RTEs varying in time. L1′s mRNAs bind to free ribosomes *r*, forming complexes *cL* with rate constant *k_bL_*; the mRNAs unbind from the complexes with rate constant *k_uL_*. Alu’s mRNAs do not translate into proteins, so Equation (4) does not contain these rates. L1′s mRNAs translate into proteins ORF1p (*O*1) and ORF2p. We assume that just synthesized ORF2p instantaneously binds to *mL* released at the end of translation, forming complex *bL* that serves as the reverse transcriptase facilitating integration of new RTE copies into the genomic DNA. Hence, no L1′s mRNAs are released at the end of translation, so Equation (3) does not contain this rate. We account for the binding of Alu’s mRNAs to ORF2p, forming complex *bS*, via the reversable reaction in which *mL* in complex *bL* is substituted by *mS*: *bL* + *mS* ↔  *bS* + *mL*. The rates of this substitution reaction with rate constants *k*_sub*S*_ and *k*_sub*L*_ for the forward and reverse reactions, respectively, are shown in Equations (3) and (4). *d_mL_* and *d_mS_* are degradation rate constants for *mL* and *mS*, respectively.

Equations (5) and (6) describe the concentration dynamics of mRNA-ribosome complexes *cq* and *cL*, respectively. The right-hand sides of the equations contain the rates of the following processes (from left to right): mRNA-ribosome complex formation, mRNA unbinding from the mRNA-ribosome complexes, complex disassembly at the finish of translation, and complex degradation.

Equations (7) and (8) describe the concentration dynamics of proteins *q* and *O*1 translated from complexes *cq* and *cL*, respectively. The equations contain the translation rates ($v_{q}$ and $v_{L}$), degradation rates with rate constants *d_q_* and *d_O_*_1_, and, in the case of Equation (8), rate $v_{\mathrm{int}L}$ of L1 integration into genome, since *O*1 is required in this process [5,23].

Equations (9) and (10) describe the concentration dynamics of complexes *bL* and *bS*, respectively. As described before, *bL* is formed at rate $v_{L}$ as a result of translation reaction from complex *cL*. It then participates in L1 integration into genome with rate $v_{\mathrm{int}L}$, in the reversable *mL*-to-*mS* substitution reaction with rate constants *k*_sub*S*_ and *k*_sub*L*_, and degrades with rate constant *d_bL_*. *bS* is reversely formed only via the *mL*-to-*mS* substitution reaction, then it promotes Alu integration into genome with rate $v_{\mathrm{int}S}$ and degrades with rate constant *d_bS_*.

Equations (11) and (12) describe the concentration dynamics of active L1 (*L*) and Alu (*S*). Their new copies are integrated into genome with rates $v_{\mathrm{int}L}$ and $v_{\mathrm{int}S}$ and degrade with rate constants $\lambda_{L}$ and $\lambda_{S}$. The RTE ‘degradation’ can be interpreted as their transition from the pool of actively transcribing RTEs to the silenced DNA regions.

There are several types of competition in the model: competition for ATP molecules in reactions proceeding with the energy consumption, competition for free ribosomes between housekeeping genes and L1′s RNAs, and competition for reverse transcriptase between L1 and Alu.

### 2.2. Reaction Rates

#### 2.2.1. Transcription

Transcription reactions and rates for mRNA production coded by housekeeping genes, L1, and Alu are as follows:

$$Q\overset{\omega_{q}\left(a\right)}{\to }Q+mq;\omega_{q}(a)=Q\cdot w_{q}\frac{a}{\theta_{q}+a}$$

$$L\overset{\omega_{L}\left(L,a\right)}{\to }L+mL;\omega_{L}(L,a)=L\cdot w_{L}\frac{a}{\theta_{L}+a}$$

$$S\overset{\omega_{S}\left(S,a\right)}{\to }S+mS;\omega_{S}(S,a)=S\cdot w_{S}\frac{a}{\theta_{S}+a}$$

The transcription rates for the RTEs depend on the number of active RTEs (*L* and *S*), which vary in time, while that for housekeeping genes depends on the fixed number *Q* of these genes. Here and everywhere below, we assume that the rates depend on the ATP concentration *a* according to the Michaelis–Menten kinetics. All parameters and their values are presented in Table 2.

#### 2.2.2. mRNA-Ribosome Complex Formation

We consider the mRNA-ribosome complex formation as a reversible reaction with the rate following the mass action law:

$$mq+f_{rib}(cq,\;cL){⇌}_{k_{uq}}^{k_{bq}}cq$$

$$mL+f_{rib}(cq,\;cL){⇌}_{k_{uL}}^{k_{bL}}cL$$

mRNAs of housekeeping genes and L1 bind to free ribosomes with concentration f_rib_(*cq*, *cL*) = *r*_tot_ − *cq* − *cL* with rate constants *k_bq_* and *k_bL_*, respectively, and unbind with rate constants *k_uq_* and *k_uL_*.

#### 2.2.3. Translation and L1′S mRNA-ORF2p Complex Formation

Proteins coded by housekeeping genes and L1 are synthesized from the mRNA-ribosome complexes. L1 RTEs code for ORF1p (*O*1) and ORF2p proteins, and ORF2p is assumed to bind L1′s mRNA immediately after translation due to the spatial proximity of the two molecules. Therefore, the translation reaction for L1 results in the formation of *O*1 and complex *bL*. Translation reactions and rates are as follows:

$$cq\overset{v_{q}\left(cq,a\right)}{\to }q;v_{q}\left(cq,a\right)=\frac{\gamma_{\max q}}{N_{q}}cq\frac{a}{K_{\gamma q}+a}$$

$$cL\overset{v_{L}\left(cL,a\right)}{\to }O1+bL;v_{L}\left(cq,a\right)=\frac{\gamma_{\max L}}{N_{L}/3}cL\frac{a}{K_{\gamma L}+a}$$

Here, $\gamma_{\max q}$ and $\gamma_{\max L}$ are the maximum translation rates in terms of the number of amino acids per min, assumed to be the same for housekeeping genes and L1 (Table 2). These values are scaled according to the protein lengths (in amino acids) to get the maximum production rates for the proteins, using an average housekeeping gene protein length $N_{q}$ and the length $N_{L}$ of the L1 genomic sequence (Table 2).

#### 2.2.4. Alu’s mRNA-ORF2p Complex Formation

As we do not consider ORF2p as a separate variable in the model, the complex *bS* consisting of Alu’s mRNA and ORF2p is modeled using a reversable reaction in which L1′s mRNA in complex *bL* is substituted with Alu’s mRNA:

$$mS+bL{⇌}_{k_{subL}}^{k_{subS}}mL+bS$$

The reaction rate follows the mass action law with rate constants *k*_sub*S*_ and *k*_sub*L*_.

#### 2.2.5. Integration of New RTE Copies into Genome

Integration of new RTE copies into the genome takes place from the transposon mRNA-ORF2p complexes, which serve as a reverse transcriptase with endonuclease activity. L1 insertions also requires ORF1p protein, which has an amino-terminal coiled-coil domain, a centrally located RNA recognition motif, and a basic carboxyl-terminal domain for normal insertions [5,23]. The insertion reactions and rates are as follows:

$$bL+O1\overset{v_{\mathrm{int}L}\left(bL,O1,a\right)}{\to }L;v_{\mathrm{int}L}\left(bL,O1,a\right)=\frac{\chi_{\max L}}{N_{L}}bL\frac{a}{K_{\chi L}+a}\cdot \frac{K_{L}O1}{1+K_{L}O1}$$

$$bS\overset{v_{\mathrm{int}S}\left(bS,a\right)}{\to }S;v_{\mathrm{int}S}\left(bS,a\right)=\frac{\chi_{\max S}}{N_{S}}bS\frac{a}{K_{\chi S}+a}$$

We account for *O*1 under the limited binding occupancy approximation. $\chi_{\max L}$ and $\chi_{\max S}$ are the maximum single-nucleotide incorporation rates in the L1 and Alu insertion reactions, respectively, assumed to be the same for the RTEs (Table 2). These values are scaled according to the length of the L1 and Alu genomic sequences ($N_{L}$ and $N_{S}$, respectively; Table 2) to get the maximum insertion rates for the full-length RTE copies.

#### 2.2.6. Energy Costs for DNA Replication

We take into account the energy costs for DNA replication considering the variation in the genome length due to new RTE insertions. The rate of energy expenditure for DNA replication is as follows:

$$v_{repl}\left(a,bL,O1,bS\right)=N_{nt}\left(\frac{N_{g}}{\tau }+N_{L}v_{\mathrm{int}L}\left(bL,O1,a\right)+N_{S}v_{\mathrm{int}S}\left(bS,a\right)\right),$$

where the number of ATP molecules required for adding one nucleotide (*N_nt_*) is multiplied by the rates of adding nucleotides to the newly synthesized DNA (in the brackets of the formula). The first rate in the brackets describes the average rate of synthesizing a genome of fixed size *N_g_* per cell cycle of duration τ. The second and third rates in the brackets correspond to the addition of new nucleotides due to new RTE insertions.

#### 2.2.7. Degradation

The degradation processes are modeled using standard first-order reactions:

$$a\overset{\lambda_{a}}{\to }\emptyset ,mq\overset{d_{mq}}{\to }\emptyset ,cq\overset{d_{cq}}{\to }\emptyset ,q\overset{d_{q}}{\to }\emptyset ,mL\overset{d_{mL}}{\to }\emptyset ,mS\overset{d_{mS}}{\to }\emptyset ,cL\overset{d_{cL}}{\to }\emptyset ,$$

$$O1\overset{d_{O1}}{\to }\emptyset ,bL\overset{d_{bL}}{\to }\emptyset ,bS\overset{d_{bS}}{\to }\emptyset ,L\overset{\lambda_{L}}{\to }\emptyset ,S\overset{\lambda_{S}}{\to }\emptyset .$$

### 2.3. Parameter Values

All variables in the model are in concentration units expressed as the number of molecules per cell (m.p.c.) for an average cell with fixed volume *V*_cell_ = 3700 μm^3^. Table 2 presents parameter values used in the model. Most values were estimated from the literature. The parameters marked with an asterisk in Table 2 were set as free parameters. We optimized their values by fitting stationary concentrations of ATP, L1, and Alu in the model to their values expected for a human cell (*a*_wt_, *L*_wt_, and *S*_wt_, respectively; Table 2). We refer to the resulted steady state as the reference stationary solution (Table 1). It represents the homeostatic energy balance in the cell in the presence of active RTEs.

**Table 2:** Parameter values in the model. bp, number of base pairs; aa, number of amino acids; m.p.c., number of molecules per cell; BNID, the entry number in the BioNumbers database of key numbers in molecular and cell biology https://bionumbers.hms.harvard.edu (accessed on 13 March 2025) [24]; UCSC Genome Browser, the University of California, Santa Cruz Genome Browser https://genome.ucsc.edu (accessed on 13 March 2025) [25]. The asterisk (*) denotes free parameters whose values were found by numerical optimization.

Designation	Description	Value	Unit	Source
*N_g_*	human genome size	3.08 × 10^9^	bp	BNID 101484
*Q*	number of housekeeping genes	3804	m.p.c.	[26]
*A*_0_	ATP influx rate	1.31 × 10^10^	m.p.c./min	BNID 110879 ^1^
*τ*	HeLa cell cycle duration	1320	min	BNID 109393
*N_q_*	median protein length for HeLa cells	431	aa	[27]
*N_Q_*	median gene length for HeLa cells	1300	bp	[27] ^2^
*N_L_*	L1 length	6000	bp	[28]
*N_S_*	Alu length	300	bp	[29]
*N_aa_*	number of ATP molecules for adding one aa	5	–	[30]
*N_nt_*	number of ATP molecules for adding one nucleotide	15	–	[30]
*χ_maxL_*	single-nucleotide incorporation rate for L1 insertion	840	1/min	[31] ^3^
*χ_maxS_*	single-nucleotide incorporation rate for Alu insertion	840	1/min	[31] ^3^
*K_χL_*	Michaelis constant of ATP consumption kinetics in L1 insertion	1.1 × 10^7^	m.p.c.	[31] ^4^
*K_χS_*	Michaelis constant of ATP consumption kinetics in Alu insertion	1.1 × 10^7^	m.p.c.	[31] ^4^
*K_L_*	L1′s mRNA–ORF1p association constant	2.24 × 10^−3^	1/m.p.c.	[32] ^5^
*w_q_*	maximum transcription rate per housekeeping gene	4.64	1/min	BNID 111721 ^6^
*w_L_*	maximum transcription rate per L1	1	1/min	BNID 111721 ^6^
*w_S_*	maximum transcription rate per Alu	20	1/min	BNID 111721 ^6^
*θ_q_*	Michaelis constant of ATP consumption kinetics in housekeeping gene transcription	3.8 × 10^9^	m.p.c.	BNID 111027 ^7^
*θ_L_*	Michaelis constant of ATP consumption kinetics in L1 transcription	3.8 × 10^9^	m.p.c.	BNID 111027 ^7^
*θ_S_*	Michaelis constant of ATP consumption kinetics in Alu transcription	3.8 × 10^9^	m.p.c.	BNID 111027 ^7^
*γ_maxq_*	maximum translation rate proteins of housekeeping genes	300	aa/min	BNID 104598
*γ_maxL_*	maximum translation rate for L1′s protein	300	aa/min	BNID 104598
*K_γq_*	Michaelis constant of ATP consumption kinetics in housekeeping gene translation	25,900	m.p.c.	[33]
*K_γL_*	Michaelis constant of ATP consumption kinetics in L1 translation	25,900	m.p.c.	[33]
*k_bq_*	housekeeping gene’s mRNA–ribosome binding rate constant	5 × 10^−8^	1/min	*
*k_bL_*	L1′s mRNA–ribosome binding rate constant	5 × 10^−8^	1/min	*
*k_uq_*	housekeeping gene’s mRNA–ribosome unbinding rate constant	0.01	1/min	*
*k_uL_*	L1′s mRNA–ribosome unbinding rate constant	0.01	1/min	*
*k_subS_*	rate constant of *mL*-to-*mS* substitution in mRNA–ORF2p complex	5 × 10^−8^	1/min	*
*k_subL_*	rate constant of *mS*-to-*mL* substitution in mRNA–ORF2p complex	5 × 10^−6^	1/min	*
*r*_tot_	total number of ribosomes	9.5 × 10^6^	m.p.c.	BNID 107347
*λ_a_*	ATP degradation rate constant	1.47	1/min	*
*d_mq_*	degradation rate constant for housekeeping gene’s mRNAs	1.15 × 10^−3^	1/min	BNID 104747
*d_cq_*	degradation rate constant for *cq* complex	1.55 × 10^−3^	1/min	*
*d_q_*	degradation rate constant for housekeeping gene’s proteins	5.67 × 10^−4^	1/min	BNID 112253
*d_mL_*	degradation rate constant for L1′s mRNAs	1.15 × 10^−3^	1/min	BNID 104747
*d_mS_*	degradation rate constant for Alu’s mRNAs	1.15 × 10^−3^	1/min	BNID 104747
*d_cL_*	degradation rate constant for *cL* complex	1.55 × 10^−3^	1/min	*
*d*_*O*1_	ORF1p degradation rate constant	5.67 × 10^−4^	1/min	BNID 112253
*d_bL_*	degradation rate constant for *bL* complex	5.67 × 10^−4^	1/min	BNID 112253
*d_bS_*	degradation rate constant for *bS* complex	5.67 × 10^−4^	1/min	BNID 112253
*λ_L_*	L1 deactivation rate constant	0.37	1/min	*
*λ_S_*	Alu deactivation rate constant	1.18	1/min	*
*V*_cell_	cell volume	3700	μm^3^	BNID 105879
*a*_wt_	number of ATP molecules in HeLa cell	5.33 × 10^9^	m.p.c.	BNID 104449
*L*_wt_	number of active L1 in human genome	1064	m.p.c.	UCSC Genome Browser
*S*_wt_	number of active Alu in human genome	13,243	m.p.c.	UCSC Genome Browser

^1^ We use an estimate for the average glucose consumption of one neuron in the human brain from this record (5.44 × 10^−9^ μmol/min) and convert it to *A*_0_ assuming that four ATP molecules appear resulting from the processing of one glucose molecule. ^2^ We assume that the median gene length in bp (*N_Q_*) is approximately three times the protein length in aa (*N_q_*). ^3^ The value was estimated from the rate-limiting step of the nucleotide incorporation kinetics in human immunodeficiency virus reverse transcription. It equals the largest value of parameter *k_p_* in Table II from ref. [31]. ^4^ The value equals the dissociation constant for dATP (5 µM) in the nucleotide incorporation kinetics in human immunodeficiency virus reverse transcription [31]. ^5^ The value equals the Rev–RRE association constant (5000 µM^−1^) in the intracellular kinetics of HIV-1, where Rev stands for control proteins that regulate the virion protein, and RRE is the Rev-responsive RNA element [32]. ^6^ The value was estimated from the maximum RNA polymerase II transcription elongation rate in HeLa cells (100 bp/sec) divided by the length of housekeeping gene (*N_Q_*). The maximum transcription rates per L1 and Alu were calculated similarly but accounting for the length of these RTEs (*N_L_* and *N_S_*, respectively). ^7^ We used the reported estimate for the transcription elongation rate of most genes in HeLa cells (3.5 Kbp/min) as an average value ${\bar{\omega }}_{q}$ and assumed the equality ${\bar{\omega }}_{q}/N_{Q}=w_{q}a_{wt}/(\theta_{q}+a_{wt})$, where *w_q_* and $a_{wt}$ were as shown in the table. Solving this equation for *θ_q_*, we found the value reported in the table. The values of *θ_L_* and *θ_S_* were found similarly, replacing *N_Q_* with *N_L_* or *N_S_* and *w_q_* with *w_L_* or *w_S_*.

### 2.4. Numerical Solution and Parameter Optimizaion

The model equations were programmed and solved in Python 3.12 using SciPy numerical integration and optimization functions. Numerical simulation was performed using the Radau method due to its high stability for stiff systems. Numerical minimization was performed using the least squares method with the Levenberg–Marquardt regularization. Linear stability of the stationary solutions was checked using the SymPy symbolic computing library.

## 3. Results

We developed the bioenergetic model (1)–(12) to theoretically study RTE activity in the context of the energy costs required for basic cellular processes. Our main focus was on finding conditions under which the energy consumption by the RTE-related processes was significant enough to shift the energy balance, thereby transferring the cell to a new state. We used the stationary solution from Table 1 as the reference steady state in this analysis. The dynamical variables in the reference state take values comparable to the available estimates for a human cell.

In what follows, we described scenarios associated with the reduction of available energy in the model to levels that potentially could initiate a cell death program. Since a drop in energy level below 30% of its reference value was reported to be incompatible with continued cell life [22], we considered this 30% energy threshold as a marker and analyzed possible ways for the energy to fall below this threshold in the model.

### 3.1. Energy Reduction Under Fixed Parameter Values

With all parameter values fixed, the only feasible way to essentially perturb energy was by varying initial conditions, especially initial number of RTEs. Firstly, new initial values of the model variables can potentially shift the dynamics to a new, lower-energy steady state, if such steady states exist. Secondly, the dynamics from the perturbed initial conditions can also reach intermediate states with lower energy before stabilization occurs. These states are also of interest depending on their duration and the amplitude of the temporary energy drop.

**Figure 2:**
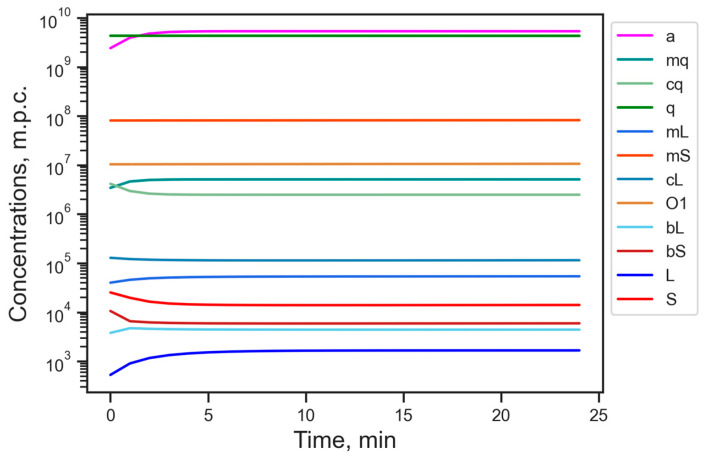
Dynamics of the model variables starting from the randomly perturbed reference state with a perturbation amplitude of up to 100%. Designations of variables are as in Table 1; m.p.c., number of molecules per cell.

The reference stationary solution appeared to be the unique stable steady state in the model under the biologically reasonable constraint of nonnegativity of all variables. As a consequence, the dynamics of all model variables asymptotically converged to the reference state even under essential initial deviations (Figure 2). Therefore, there was no way to shift the stationary energetic balance in the cell at the fixed parameter values.

**Figure 3:**
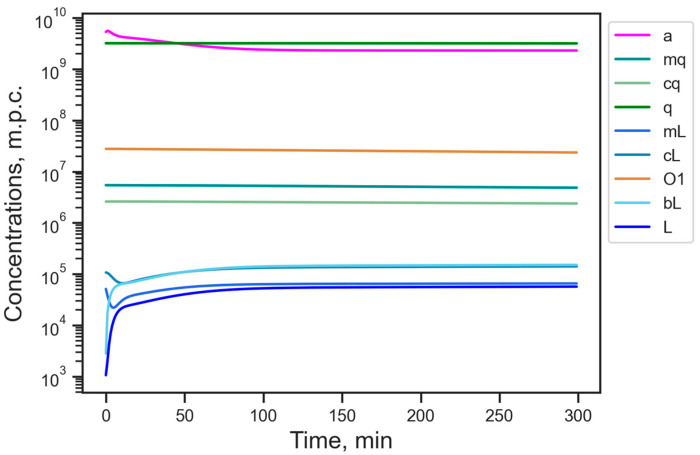
Dynamics starting from the reference steady state (Table 1) in the reduced model, in which Alu and its molecular products are absent. Designations of variables are as in Table 1; m.p.c., number of molecules per cell.

It is interesting that a reduced version of the model in which Alu is completely absent (*S* = 0) has a steady state with the level of available energy 2.3 lower than the reference value from the full model. Since Alu competes with L1 for the retrotransposition machinery, the absence of this competition in the reduced model leads to the dynamics converging to an elevated stationary number of L1 compared to the reference state (Figure 3). The dynamics also exhibit an increased stabilization time for the ORF1p protein concentration (*O*1), which significantly exceeds a typical cell lifetime. These effects show that the activity of Alu in the cell holds L1 from excessive abundance. It supports the vitality of the cell by implicitly reducing the energy burden and, thus, acting as an additional defense mechanism. However, since Alu and L1 are both typically active in cancer cells, we will not consider the reduced model further.

**Figure 4:**
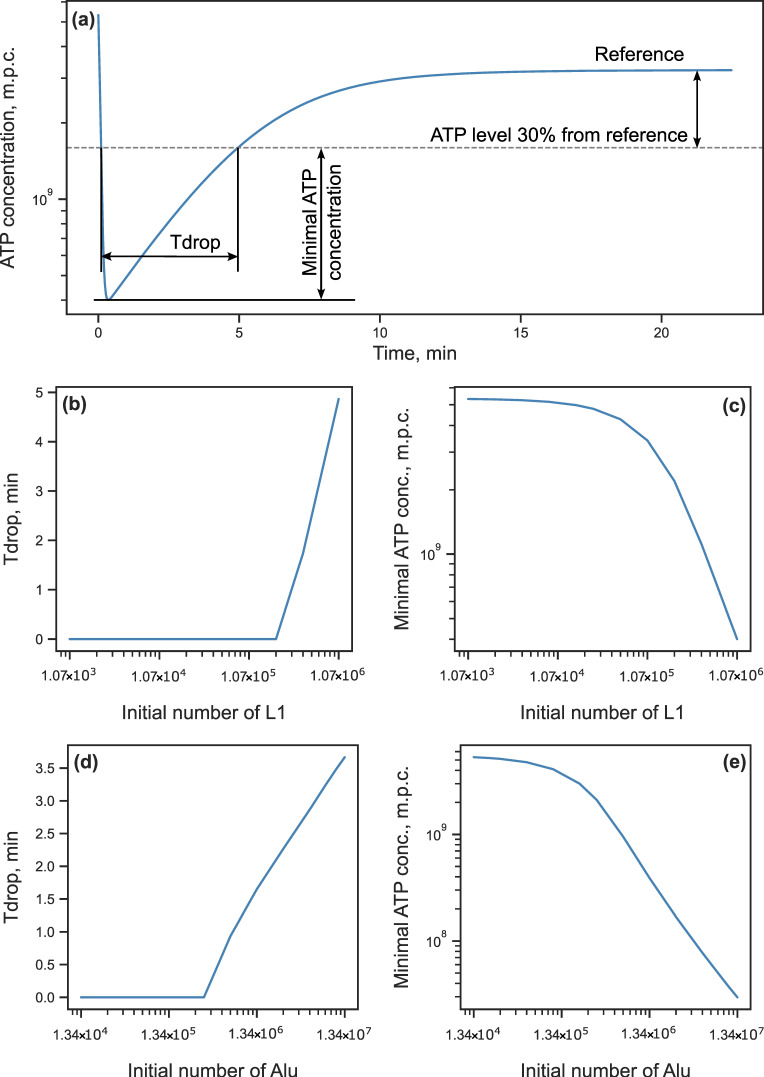
Temporary energy reduction in response to increasing numbers of L1 and Alu in the genome. (**a**) Illustration of how the duration (*T*_drop_) and depth (‘minimal ATP concentration’) of the energy drop are determined using the example of the ATP concentration dynamics for the initial L1 number increased 1000-fold compared to its reference value (1064 copies, Table 1); (**b**) Dependence of *T*_drop_ on the initial L1 number; (**c**) Dependence of the minimal ATP concentration on the initial L1 number; (**d**,**e**) Same as (**b**,**c**), respectively, but for the dependence on the initial Alu number, which has the reference value equal to 1.34 × 10^4^ (Table 1).

Despite the inability to reduce the stationary ATP level by varying the initial conditions, we can achieve this for some period before the dynamics stabilizes. A feasible way to perturb the initial cellular state consists in varying the number of L1 and Alu in the genome, which can be accomplished in practice by editing the genome with an RTE containing construct insertion. We investigated how changes in the initial number of RTEs affected the energy level prior to stabilization by simulating the model solution from an initial state with modified numbers of RTEs and reference values of all other model variables. We determined the duration of the energy drop below the 30% threshold and the maximum depth of the drop in the dynamics as the RTE influence efficiency measures (Figure 4a). The simulations showed that the ATP concentration dropped below the threshold starting at approximately 100-fold increased L1 numbers (Figure 4b,c) or 20-fold increased Alu numbers (Figure 4d,e). When the L1 number was increased to 1000-fold the reference value, the energy dropped by more than an order of magnitude for about 5 min (Figure 4b,c). When the Alu number was increased in the same proportion, the duration of the energy drop was smaller (around 4 min), but the magnitude of the drop was larger (Figure 4d,e). These results demonstrate that we can indeed influence the cellular energy at the fixed parameter values by varying the number of RTEs in the genome. However, this influence would require a large number of new RTE insertions, and the effect would not last very long.

### 3.2. Energy Reduction Under Perturbed Parameter Values

#### 3.2.1. Parameter Sensitivity Analysis

A more efficient scenario for shifting the energetic balance away from the reference state is to accompany the RTE number variation with additional modifications of cellular processes that would help shift the cell away from the reference conditions. To find such processes among those parameterized in our model, we performed the parameter sensitivity analysis in the model and analyzed parameters whose variation led to significant energy changes. We calculated the response coefficients for the energy, which are partial derivatives of the energy *a* with respect to each parameter in the model at the reference steady state. These are linear sensitivity coefficients quantifying the magnitude of the energy response to small perturbations of parameter values in a vicinity of the reference steady state.

**Figure 5:**
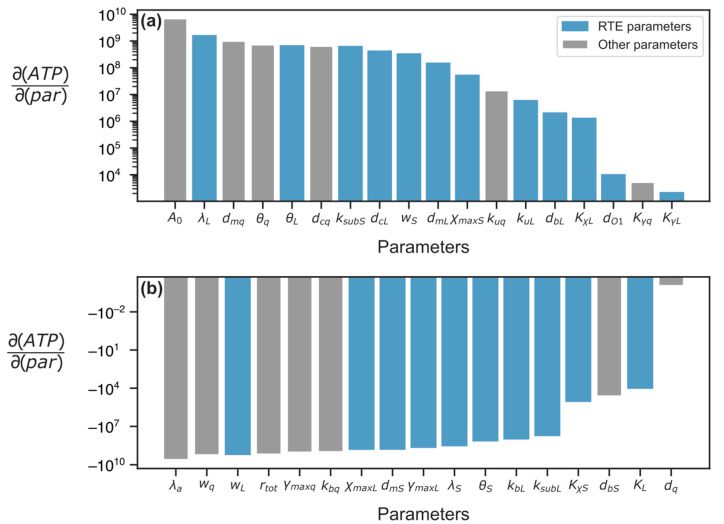
Energy response coefficients for the model parameters exerting (**a**) positive and (**b**) negative impact on the ATP concentration near the reference steady state. Blue color indicates the parameters associated with RTEs, and grey color marks other parameters. Parameters associated with housekeeping genes contain ‘q’ in their subscript. Definitions of all parameters are given in Table 2.

Arranging parameters according to the response coefficient values, we found that the energy influx rate (*A*_0_) and ATP degradation rate constant (*λ_a_*) had the greatest impact on the energy level (Figure 5). Among the RTE-related parameters, the energy level is most sensitive to the L1 maximum transcription rate (*w_L_*) and the rate constant *λ_L_* of the L1 degradation. The RTE degradation in the model can be interpreted as the RTE deactivation process, associated, for example, with transition to the non-transcribed chromatin regions.

**Figure 6:**
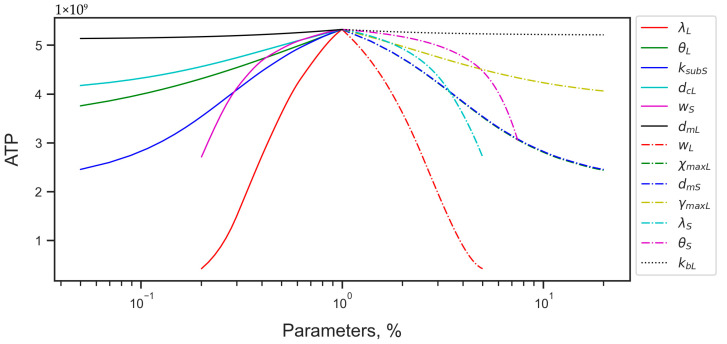
Steady-state ATP level under perturbed parameter values. Each curve represents the ATP number in the steady state under the perturbation of a parameter shown in the inset. The perturbation magnitude is shown in the horizontal axis as a proportion of the reference value. The central point in the horizontal axis corresponds to the reference parameter value. The curves to the left of the reference value correspond to the perturbation of parameters with the positive energy response (larger parameter values lead to larger ATP numbers), and ones to the right, to the negative response (larger parameter values lead to smaller ATP numbers).

We additionally investigated the sensitivity of the stationary energy level to larger perturbations of the RTE-related parameters, where the linear analysis stopped being informative. The same parameters as for the linear analysis (*λ_L_* and *w_L_*) appeared to exert the strongest impact on the energy in a wide parameter value range (Figure 6). The nonlinear sensitivity analysis also revealed that the order of parameters with respect to their contribution to energy reduction could change as the perturbation magnitude increased. In particular, the Alu deactivation rate constant *λ_S_* and the Michaelis constant *θ_S_* in the energy dependence of the Alu transcription rate become more prominent in the influence on the ATP level at large perturbations among the negative-response parameters (Figure 6). Among the positive-response parameters, the Alu maximum transcription rate *w_S_* becomes the second most influential under large perturbations (Figure 6).

#### 3.2.2. Energy Reduction and Redistribution Among the Processes Under Perturbations of RTE-Associated Parameter Values

**Figure 7:**
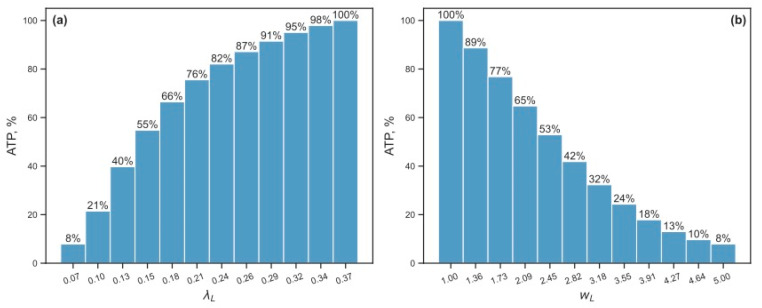
A drop in the steady-state ATP level (in percentage from the reference value) in response to the variation of (**a**) the L1 deactivation rate constant *λ_L_* and (**b**) the L1 maximum transcription rate *w_L_*.

**Figure 8:**
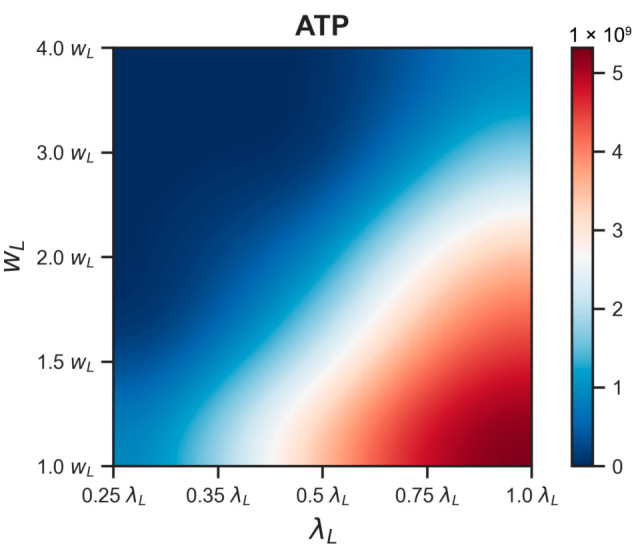
Steady-state ATP level under combined variation of parameters *λ_L_* and *w_L_*. The lower right corner corresponds to the reference state.

A more detailed picture of how the stationary ATP level declines relative to the reference in response to changes in *λ_L_* or *w_L_* shows that the energy dropping curve is steeper when *w_L_* increases (Figure 7). Changing the values of these parameters by 3 times from their reference values resulted in a decrease in the stationary ATP level to 30% of its reference value. The combined variation of *λ_L_* or *w_L_* led to even more efficient energy reduction (Figure 8). It was sufficient to change the parameter values by √2 times less when changing *λ_L_* and *w_L_* simultaneously than separately to achieve the same energy reduction.

**Figure 9:**
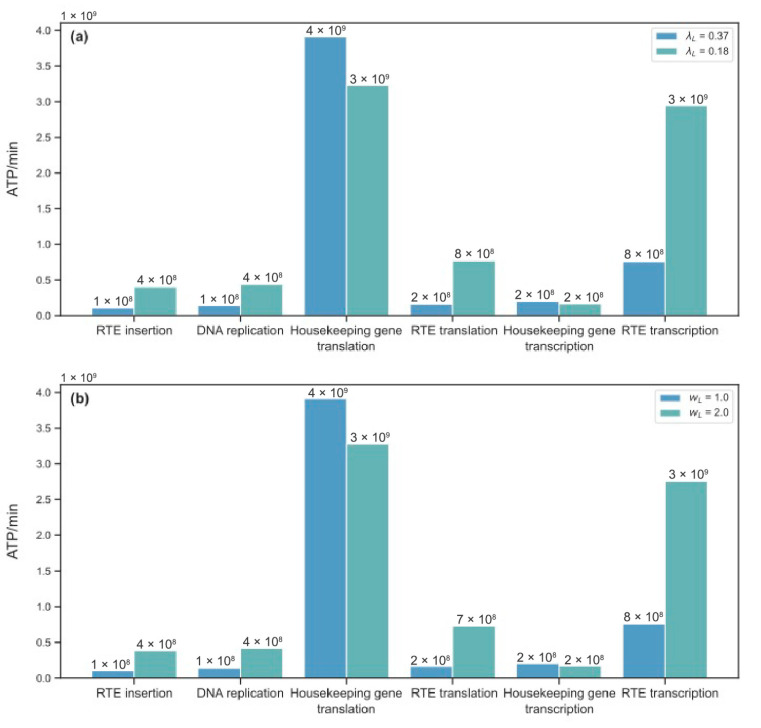
Energy consumption by various cellular processes. Blue color marks the energetic costs in the reference steady-state, and green color corresponds to the steady-state costs under perturbed values of (**a**) *λ_L_* and (**b**) *w_L_*.

The described decrease in free ATP levels was associated with a redistribution of cellular energy among the cellular processes. A significant shift occurred towards a higher ATP consumption by RTE-related processes (Figure 9). Translation was the main energy consuming cellular process at the reference state in the model (as expected [34]) and showed an essentially reduced energy consumption rate under the modified parameters *λ_L_* and *w_L_*. The RTE transcription demonstrated the largest increase in energy consumption under the modified conditions. Its consumption rate approached to that for housekeeping protein translation. Other processes with the increased energetic costs included transposon translation and insertion into the genome, as well as DNA replication, since a larger number of new RTE copies resulted in the increased DNA length.

## 4. Discussion

We presented a new dynamical model that describes how the activity of RTEs influences the energetic balance in the cell. The energy-consuming processes were described in the model using coarse-grained approximations, following a similar approach previously applied for prokaryotic cells [33,35]. Our modeling approach added a new bioenergetic context to studies of genomic RTE dynamics as compared to previous models [6,36,37,38,39,40,41,42]. In contrast to the population approach common in previous studies, we considered the ‘average’ cell under different metabolic conditions. Our model also reproduced the predator–prey nature of the dynamical competition between L1 and Alu shown previously [6,42], but included more molecular details of this competition.

The distribution of energy costs among the main cellular processes in the reference steady state in the model corresponded to the expected distribution for a eukaryotic cell. In particular, translation was the leading energy consuming process, while transcription and other processes provided much less energetic burden [34]. The steady state concentrations of molecules considered in the model, including cellular ATP levels and RTE counts, were also in the range of values previously reported (Table 2).

A conventional way of studying the interplay between mobile elements and oncogenesis is through the investigation of their involvement in various metabolic and signaling pathways within cancer cells [9,43]. We applied an alternative approach and used our bioenergetic model to explore the evolution of LINE-1 and SINE retrotransposons in the context of their influence on the total level of free ATP in the cell. ATP level was shown to be a good marker associated with the cell death program initiation [20,21,22]. We inferred two basic scenarios in which ATP levels were shifted towards critical values by modulating the RTE activity.

In both scenarios, higher numbers of actively proliferating L1 and Alu elements in the genomic DNA led to energy depletion in the cell. The direct practical way to increase these numbers is to insert multiple new RTE copies into the DNA. In the context of the model, this action corresponds to increasing the initial ‘wild type’ values of the RTE counts (1064 L1 elements and 13,243 Alu elements). Simulations showed that this scenario could reduce the cellular ATP levels for short time periods only, since the steady state in the model was the global attractor for biologically reasonable concentration values of all molecules. The amplitude and duration of the temporary energy drop was dependent on the initial RTE counts.

The second way to fuel the production of new active RTE copies is to modify the rates of processes associated with the RTE life cycle. The sensitivity analysis and simulations showed that the L1 maximum transcription rate and L1 deactivation rate constant were the best targets for variations that could reduce ATP levels down to critical values. Parameters associated with Alu reproduction were effective for energy reduction only under large variations of their values. The parameter variation may lead to the essential redistribution of energy costs over the cellular processes. In particular, costs for RTE transcription and translation may become of the same order as translation for all other genes.

The presented scenarios of creating an energy deficit in cancer cells due to the activation of mobile elements require further experimental verification. Our modeling results suggest that the most effective way to influence the bioenergetic balance using RTEs is a combination of the two scenarios described above. However, these scenarios demand different practical efforts. The initial variation of active L1 and Alu in the genomic DNA is a feasible approach, which can be achieved by cell transfection with a plasmid containing a genetic construct with active RTE copies [44]. As the model predicts only temporal variations of ATP levels in this case, it would be interesting to investigate the reliability of the cell death program initiation in response to ATP level variations of various amplitudes and duration.

In contrast to the first scenario, it seems problematic to modify the RTE-associated process rates in practice without possible negative effects accompanying such modifications. For example, perturbation of the RTE deactivation rate constants (*λ_L_* and *λ_S_*) can be performed by demethylating DNA and, thus, opening a larger DNA segment for active RTE accumulation. However, hypomethylation may have additional, RTE-independent consequences for cellular processes, which can both influence the bioenergetics and trigger molecular pathways that would be hard to identify or decouple from the pure action of RTEs. To account for such experimental data, a more elaborate (less coarse-grained) model that incorporates more cellular details is probably required. We believe the presented model can serve as a first step towards this goal. The presented analysis of this model can be considered as a theoretical ‘proof-of-concept’ in developing anticancer strategies based on the controlled RTE influence on cellular energetics.

### Model Limitations and Possible Future Research Directions

Several factors determined the theoretical nature of our study. The only experimental information implemented in the model was based on the literature estimates of the parameter values. Despite many parameter values in the model were related to HeLa cells, the cell under modeling should be considered as a rather ‘typical’ eukaryotic cell, since not all information was available for a single cell type. In particular, some degree of freedom in the presented modeling approach was reflected in the steady-state expression levels, which resulted from the parameter optimization. RNA-seq data could be an alternative source for the quantification of RTE and/or housekeeping gene expression levels [45].

Different cell types may vary in the RTE expression levels. L1 and Alu activity in HeLa cells was confirmed and investigated in detail [46,47]. HeLa cells, along with certain other epithelial cancer lines, exhibit a unique three-dimensional genome architecture that allows them to tolerate elevated levels of RTE transcripts. In contrast, myeloid leukemia cells significantly downregulate LINE-1 expression via SETDB1-mediated H3K9 trimethylation, likely preventing activation of the interferon pathway [48]. To make cell-specific predictions, the parameter values in the model should be adjusted to the cell type of interest, with possible subsequent variation in the energy reduction scenarios compared to those predicted in our study.

Our model is rather simple and does not include many molecular details. Mutational processes render the vast majority of L1 copies in the genome inactive and make ORF1 and ORF2 sequences functionally lost [49,50,51]. This effect can be taken into account by tuning the effective values of the degradation constants for the proteins and RTEs, but a refinement of the transcription, translation, and complex formation mechanisms in the equations is a possible alternative. Another aspect concerns the fact that the model considers only the full-length L1, while most of the L1 copies in the genome are truncated upstream of their 3′ ends [52]. Consequently, it is possible that the model overestimates the expression level of L1 resulted from the changes in L1 copy number. The model can be modified to directly account for the truncated L1 copies, or more advanced modeling techniques can be applied [40].

We did not examine in detail the specific metabolic pathways that directly initiate programmed cell death, nor did we incorporate immune system responses explicitly. This corresponds to the assumption that the energy consumption associated with these processes can be neglected or attributed to the effective value of the degradation constant for the energy. An improved model integrating immune signaling pathways will enhance the biological fidelity of the model and allow to accurately match the energy load of RTEs with their effects through the interplay with the immune system.

Future refinements may include feedback loops between p53 and L1, in which p53 loss leads to enhanced retrotransposon activity, resulting in increased genomic instability and a subsequent drop in cellular energy levels [53,54,55]. A simple mathematical model was applied to elucidate the dual role of p53 in regulating both L1 expression and retrotransposition [56]. Similarly, BRCA1, a protein involved in repairing damaged DNA, has been shown to promote L1 RNA processing and inhibit its nuclear export; thus, BRCA1 deficiency could further stimulate retrotransposition [57]. The oncogene KRAS is also known to reprogram metabolism by upregulating glycolysis and altering chromatin structure [58]. Additionally, future studies may explore how hypoxia and oxidative stress, which are the common features of the tumor microenvironment, modulate L1 expression and transposon activity.

## Figures and Tables

**Table 1:** Dynamical variables in the model and their values in the reference stationary solution. m.p.c., number of molecules per cell.

Designation	Dynamical Variable	Stationary Value, m.p.c.
*a*	amount of available energy (ATP level)	5.32 × 10^9^
*mq*	mRNA of housekeeping genes	5.44 × 10^6^
*cq*	complex of *mq* with ribosomes	2.61 × 10^6^
*q*	protein translated from *cq*	3.20 × 10^9^
*mL*	mRNA of L1	5.13 × 10^4^
*mS*	mRNA of SINE	1.22 × 10^8^
*cL*	complex of *mL* with ribosomes	1.8 × 10^5^
*O*1	ORF1p protein translated from *cL*	2.78 × 10^7^
*bL*	complex of ORF2p proteins with *mL*	2.83 × 10^3^
*bS*	complex of ORF2p proteins with *mS*	5.64 × 10^3^
*L*	number of L1 in genome	1.07 × 10^3^
*S*	number of Alu in genome	1.34 × 10^4^

## Data Availability

The code implementing the model can be downloaded from the Zenodo repository: https://doi.org/10.5281/zenodo.15022049.
